# A thiadiazolylidene-morpholine compound inhibits *Pseudomonas aeruginosa* by destabilizing the thiamine monophosphate kinase thiL

**DOI:** 10.1016/j.jbc.2026.113112

**Published:** 2026-05-06

**Authors:** Yingying Li, Jianqing Lin, Zara Chung, Benny Ken Yee Yeo, Julien Lescar, Kevin Pethe

**Affiliations:** 1 Lee Kong Chian School of Medicine, Nanyang Technological University, Singapore; 2 NTU Institute of Structural Biology, Nanyang Technological University, Singapore; 3 School of Biological Sciences, Nanyang Technological University, Singapore; 4 Antimicrobial Resistance Interdisciplinary Research Group, Singapore-MIT Alliance for Research and Technology Centre, Singapore; 5 Singapore Centre for Environmental Life Sciences and Engineering (SCELSE), Singapore; 6 Ineos Oxford Institute for Antimicrobial Research (IOI), Department of Biology, University of Oxford, Oxford, UK

**Keywords:** antibacterial inhibitor, metabolic vulnerability, protein destabilization, *Pseudomonas aeruginosa*, thiamine monophosphate kinase (ThiL)

## Abstract

*Pseudomonas aeruginosa*, an opportunistic gram-negative pathogen, poses a growing threat in healthcare-associated infections. Its intrinsic resistance and acquisition of carbapenemases have driven widespread multidrug resistance and severely limited treatment options. *P. aeruginosa* causes life-threatening infections including ventilator-associated pneumonia, bloodstream infections, complicated urinary tract infections, and chronic lung disease in cystic fibrosis. We identified and validated *thiL*, encoding thiamine monophosphate kinase, as a critical metabolic vulnerability and promising antibacterial target. ThiL deletion abolished virulence in murine lung and wound models and rendered bacteria incapable of survival without a supraphysiological level of thiamine pyrophosphate. A screen of 1231 kinase inhibitors identified VP3.15 as the first specific ThiL inhibitor with antibacterial potency. Mechanistic studies showed VP3.15 destabilizes ThiL, promoting protein unfolding and functional loss. These results establish ThiL as a druggable target and highlight metabolic dependencies as a therapeutic opportunity against multidrug-resistant *P. aeruginosa*.

*Pseudomonas aeruginosa* is a ubiquitous gram-negative bacterium and a leading cause of hospital-acquired infections worldwide (1). This opportunistic pathogen is responsible for a wide range of severe and often life-threatening infections, including ventilator-associated pneumonia, bloodstream infections, complicated urinary tract infections, burn wound infections, and chronic pulmonary infections in individuals with cystic fibrosis and bronchiectasis (1, 2). Its remarkable environmental adaptability, intrinsic resistance to multiple classes of antibiotics, and ability to form biofilms contribute to its persistence and significant clinical burden. Treatment has become increasingly difficult due to its intrinsic resistance mechanisms and its exceptional ability to acquire additional determinants, including reduced outer membrane permeability, active efflux pumps, and the production of antibiotic-modifying enzymes (3). Particularly concerning is the global rise of multidrug-resistant and carbapenem-resistant *P. aeruginosa*, which severely limits therapeutic options and is associated with increased mortality and prolonged hospital stays (4). The World Health Organization has classified carbapenem-resistant *P. aeruginosa* as a critical priority pathogen, underscoring the urgent need for new antibacterial strategies that circumvent conventional resistance mechanisms.

One promising alternative lies in the targeting of essential bacterial metabolic pathways. Unlike traditional antibiotic targets such as cell wall, protein, or DNA synthesis, metabolic enzymes that are indispensable for bacterial growth and virulence constitute attractive targets (5). Metabolic enzymes represent an underexploited class of vulnerabilities that are indispensable for bacterial growth and virulence (5). Disruption of key metabolic nodes not only affects replication but can also attenuate virulence, colonization, and persistence within the host. Examples such as the folate pathway (6), NAD biosynthesis (7), and menaquinone biosynthesis (8, 9) have validated metabolic enzymes as effective antibacterial targets.

However, compared to these well-studied pathways, vitamin biosynthesis remains far less explored, even though vitamins provide essential cofactors for central enzymatic reactions and are often difficult for pathogens to bypass. Among vitamins, thiamine (vitamin B1) metabolism is of particular interest. Its active cofactor, thiamine pyrophosphate (TPP), is indispensable for multiple reactions in central carbon metabolism and energy generation, including pyruvate dehydrogenase, α-ketoglutarate dehydrogenase, branched-chain ketoacid dehydrogenase, and transketolase (10, 11). These enzymes connect glycolysis, the tricarboxylic acid cycle, and the pentose phosphate pathway, underscoring the centrality of TPP to bacterial physiology, thereby positioning thiamine metabolism as a relevant target for antimicrobial development.

The therapeutic promise of this strategy lies in fundamental nutritional differences between pathogens and their human hosts. Humans must obtain thiamine from their diet and directly phosphorylate it to TPP *via* thiamine pyrophosphokinase (12). In contrast, most gram-negative bacteria possess a complete *de novo* biosynthetic pathway (13, 14). The final and essential step in the pathway is catalyzed by thiamine monophosphate kinase (ThiL), which converts the intermediate thiamine monophosphate (TMP) into TPP.

Importantly, this step is absent in human metabolism, where TMP is not a requisite precursor for TPP synthesis. Given that humans lack both *de novo* thiamine synthesis and a functional ThiL ortholog, selective inhibition of ThiL is predicted to disrupt TPP homeostasis in pathogens with minimal mechanism-based toxicity to the host.

The genetic essentiality of ThiL in key gram-negative pathogens, supported by earlier studies, confirms this metabolic distinction as a promising therapeutic vulnerability. We previously demonstrated that oxythiamine functions as a potent antimetabolite in *P. aeruginosa*, interfering with thiamine metabolism and impairing bacterial growth (15). Furthermore, Jang *et al*. characterized the *P. aeruginosa* ThiL and reported inhibitors of the enzyme (16). However, whether ThiL represents an essential and druggable vulnerability in the cellular and *in vivo* context has not been fully established, and none of the reported inhibitors have yet been validated as acting through direct on-target engagement in bacterial cells.

Building on these insights, we provide additional genetic and pharmacological validation of thiL as a critical metabolic vulnerability in *P. aeruginosa*. By integrating biochemical, microbiological, and *in vivo* infection models, we demonstrated that ThiL is indispensable for growth and pathogenesis and identified VP3.15 as the first small-molecule inhibitor with clear evidence of cellular target engagement. Our work thus establishes ThiL as a tractable drug target in gram-negative bacterium and highlights the potential of metabolic pathways as an untapped source of antibacterial targets.

## Results

### *P. aeruginosa* is unable to efficiently utilize exogenous TPP

**Figure 1:**
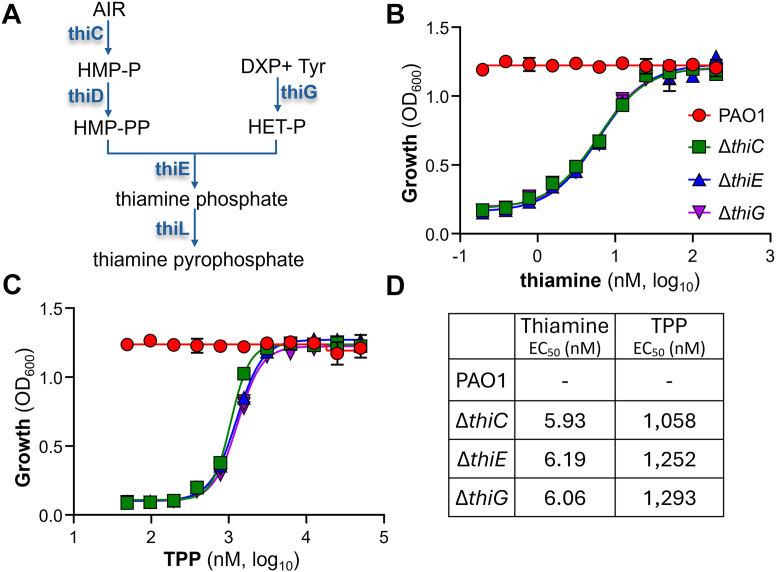
**Loss of *de novo* vitamin B1 biosynthesis renders *P. aeruginosa* auxotrophic for thiamine.***A*, *P. aeruginosa de novo* vitamin B1 biosynthetic pathway. *B* and *C*, growth curves of PAO1, Δ*thiC*, Δ*thiE*, and Δ*thiG* in minimal medium supplemented with varying concentrations of thiamine (*B*) or TPP (*C*). Bacterial growth was assessed by measuring absorbance at 600 nm (OD_600_) after 20 h of incubation. *D*, half-maximal effective concentrations (EC_50_) of thiamine and TPP for PAO1, Δ*thiC*, Δ*thiE*, and Δ*thiG* using nonlinear regression analysis of dose-response curves. Data represent mean ± SD of three independent experiments. AIR, 5-aminoimidazole ribonucleotide; DXP, 1-deoxy-D-xylulose phosphate; HMP-P, 4-amino-2-methyl-5-hydroxymethyl pyrimidine monophosphate; HMP-PP, 4-amino-2-methyl-5-hydroxymethyl pyrimidine diphosphate; TPP, thiamine pyrophosphate; Tyr, L-tyrosine.

To evaluate the potential of *thiL* as a drug target, we first examined whether *P. aeruginosa* can efficiently utilize exogenous TPP. If the bacterium were capable of high-affinity TPP uptake, inhibition of ThiL would be readily bypassed, limiting its therapeutic value. We therefore generated *thiC*, *thiE*, and *thiG* deletion mutants *via* homologous recombination to disrupt the *de novo* thiamine biosynthesis pathway and probe thiamine and TPP transport (Fig. 1*A*). As expected, all three mutants exhibited thiamine auxotrophy, confirming their inability to synthesize thiamine. Supplementation with thiamine restored growth with half-maximal effective concentration (EC_50_) values between 5.93 and 6.19 nM (Fig. 1, *B* and *D*), consistent with the presence of a high-affinity thiamine uptake system. In contrast, supplementation with TPP failed to efficiently rescue growth; EC_50_ values for TPP ranged from 1058 to 1293 nM (Fig. 1, *C* and *D*), one order of magnitude higher than physiological concentrations found in human tissues (17). These findings demonstrate that *P. aeruginosa* lacks an efficient TPP uptake mechanism, rendering intracellular TPP synthesis *via* ThiL indispensable and for survival. By comparison, enzymes involved upstream in the *de novo* thiamine biosynthetic pathway represent less promising antibacterial targets, as their inhibition can be bypassed by environmental thiamine uptake through high-affinity transporters.

### ThiL is essential for bacterial growth and for virulence in lung and wound infection models

**Figure 2:**
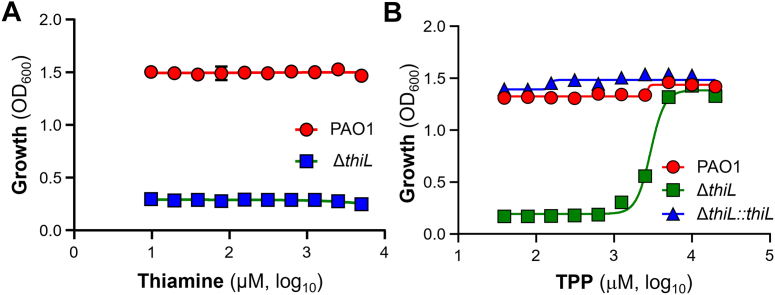
**TPP, but not thiamine, rescues growth of Δ*thiL* mutant.***A*, growth of wild-type PAO1 and the Δ*thiL* mutant in M9 minimal medium supplemented with increasing concentrations of thiamine. *B*, growth of PAO1, Δ*thiL*, and complement in M9 minimal medium supplemented with increasing concentrations of TPP. Bacterial growth was assessed by measuring absorbance at OD600 after 20 h of incubation. Data represent mean ± SD of three independent experiments. TPP, thiamine pyrophosphate.

**Figure 3:**
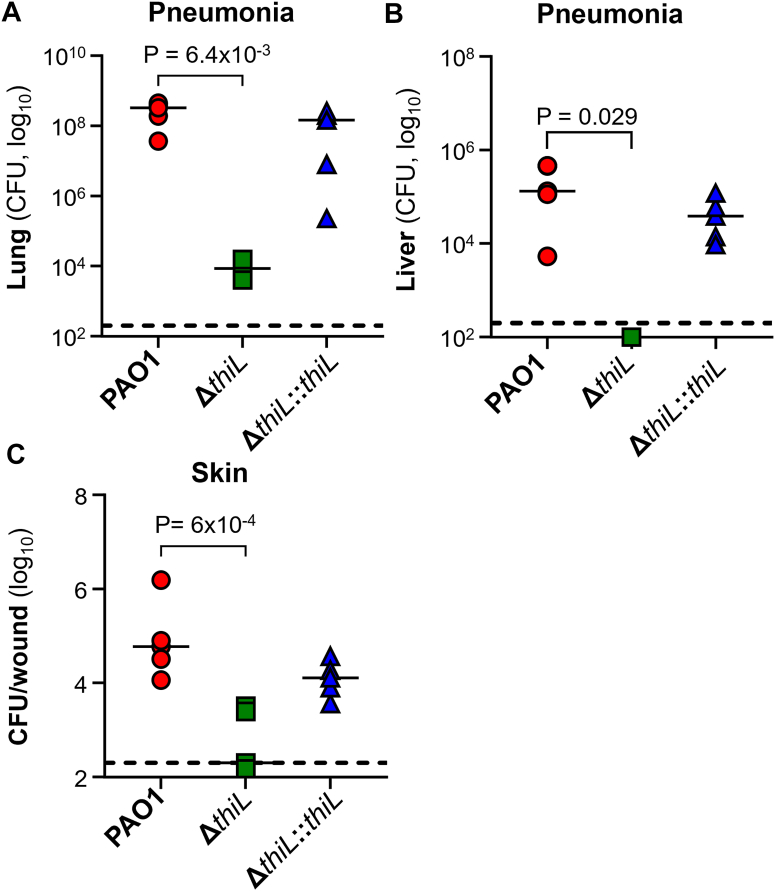
**ThiL is required for lung and wounds infection.** Neutropenic mice were infected by the PAO1, PAO1Δ*thiL*, or PAO1Δ*thiL*::*thiL* strains by the intranasal route. Bacterial burden was determined in the lung (*A*) and liver (*B*) 15 h postinfection. *C*, wound of immunocompetent mice were infected with the PAO1, PAO1Δ*thiL*, or PAO1Δ*thiL*::*thiL* strains. Skin samples were collected 24 h postinfection, and bacterial loads were quantified by CFU determination on agar plates. Data represent mean ± SD of five mice per group. Statistical analysis was performed using one-way ANOVA followed by Tukey’s multiple comparisons test. ThiL, thiamine monophosphate kinase.

To directly assess the essentiality of ThiL, we constructed a *thiL* deletion mutant under conditions supplemented with high concentrations of exogenous TPP. The mutant was viable only when grown in the presence of ∼5 mM TPP (Fig. 2*B*), a concentration nearly 50,000-fold higher than physiological TPP levels (17), underscoring the negligible passive uptake of TPP and reinforcing the critical requirement for ThiL. As expected, the PAO1Δ*thiL* strain was unable to grow when supplemented with thiamine (Fig. 2*A*). Growth was fully restored by episomal *thiL* expression (Fig. 2*B*), confirming that the observed phenotype was directly attributable to loss of ThiL. Consistent with previous work demonstrating that PAO1Δ*thiL* is severely attenuated in an immunocompetent murine lung infection model, we observed a comparable impairment in bacterial replication in the lungs (Fig. S1*A*). We extended these findings by showing that the mutant was also unable to disseminate to distal organs, including the liver (Fig. S1*B*). Moreover, because *P. aeruginosa* most frequently causes infections in immunocompromised hosts, we evaluated PAO1Δ*thiL* in a neutropenic lung infection model, where it displayed drastic attenuation of lung replication (Fig. 3*A*) and near-complete failure to disseminate to the liver (Fig. 3*B*) and spleen (Fig. S1*C*). Finally, we showed that ThiL is essential beyond pulmonary infection, as PAO1Δ*thiL* also exhibited severely restricted proliferation in a murine skin infection model (Fig. 3*C*). Together, these results establish ThiL as a central determinant of *P. aeruginosa* pathogenesis across multiple host environments.

### Identification and validation of VP3.15 as a ThiL inhibitor with antibacterial potency

**Figure 4:**
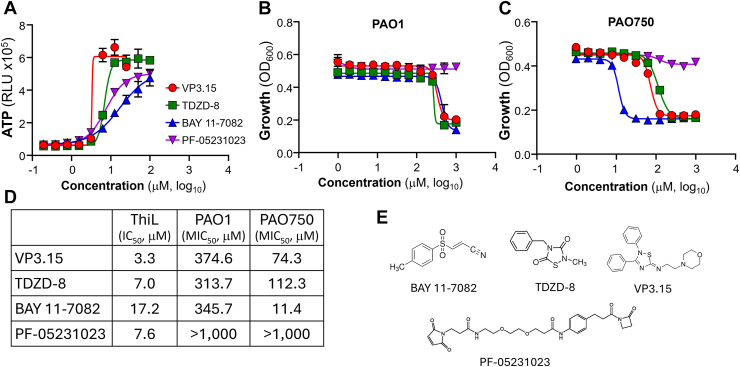
**Inhibitory activity of kinase inhibitors against ThiL and *P. aeruginosa*.***A*, inhibitory potency of four hits against recombinant ThiL. *B*, growth curves of parental PAO1 with increasing concentrations of the hits. *C*, growth curves of the efflux pump mutant strain PAO750 exposed to the same hits. *D*, MIC_50_ of the four hits against PAO1 and PAO750, determined by nonlinear regression of dose-response data. *E*, chemical structure of the hits. ThiL, thiamine monophosphate kinase.

**Figure 5:**
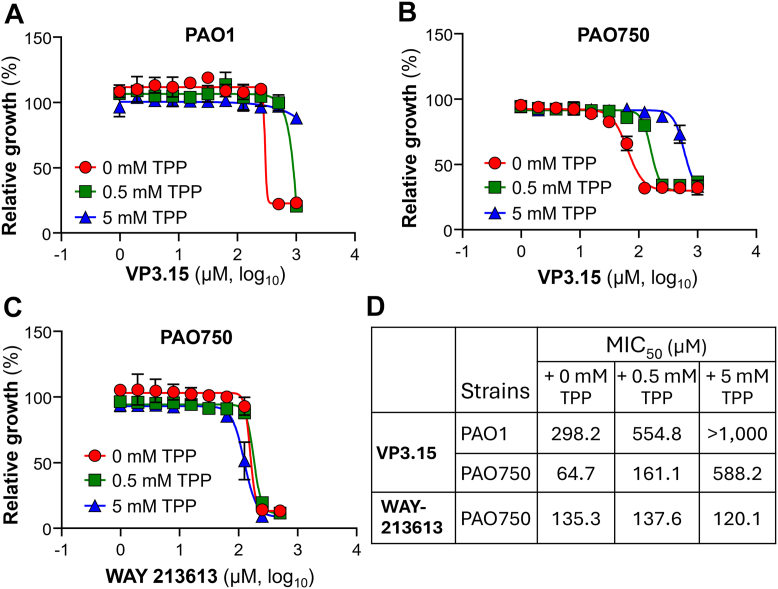
**Effect of exogenous TPP on the growth inhibitory potency of VP3.15 and WAY-213613.** Inhibitory potency of VP3.15 against PAO1 (*A*) and PAO750 (*B*) in the presence of 0, 0.5, or 5 mM TPP. Bacterial growth was monitored by OD600. *C*, inhibitory potency of WAY-213613 against PAO750 in the presence of 0, 0.5, or 5 mM TPP, measured by OD600. *D*, MIC_50_ of VP3.15 and WAY-213613 in the presence of varying concentrations of TPP. Values were determined based on nonlinear regression of dose-response curves. TPP, thiamine pyrophosphate.

Given the essentiality of ThiL, we developed a target-based enzymatic assay to identify small-molecule inhibitors. Recombinant *P. aeruginosa* ThiL was expressed in *Escherichia coli* and purified (Fig. S2*A*). The assay was optimized for linear reaction kinetics and maximal signal-to-background ratios. Robustness was confirmed with a Z′-factor of 0.88, calculated under ATP-present (positive) or ATP-absent (negative) conditions. Enzyme activity was unaffected by dimethyl sulfoxide up to a concentration of 2% (Fig. S2*B*), ensuring compatibility with compound library screening. A focused set of 1231 kinase inhibitors was screened at a concentration of 10 μM, yielding four candidates with IC_50_ values between 3.32 and 17.2 mM (Fig. 4, *A* and *D*). Three compounds inhibited growth *of P. aeruginosa* PAO1, with MIC_50_ values ranging from 313.7 to 374.6 μM (Fig. 4, *B* and *D*). To assess whether limited intracellular accumulation restricted potency, MICs were determined in the efflux-deficient strain PAO750 (18). All three compounds displayed markedly enhanced activity in this background, with ∼2- to 30-fold lower MICs compared to PAO1 (Fig. 4*C*). Among these, only VP3.15 showed TPP-reversible growth inhibition in both PAO1 (Fig. 5*A*) and PAO750 (Fig. 5*B*), confirming that its antibacterial effect is attributable to ThiL inhibition. By contrast, TPP supplementation did not alter the antibacterial activity of TDZD-8 and BAY 11 to 7082, suggesting that their effects occur through off-target mechanism(s). Previously, Kim *et al.* identified WAY-213613 as a ThiL inhibitor with an *in vitro* Ki of 13.4 mM and reported growth inhibition only when combined with subinhibitory colistin (16). We confirmed that WAY-213613 was inactive against PAO1 (MIC_50_ > 1000 μM) but showed potency against PAO750 at an MIC_50_ of 135 μM, indicating efflux-limited activity. However, unlike VP3.15, the potency of WAY-213613 was not alleviated by exogenous TPP (Fig. 5*C*), demonstrating that growth inhibition is unrelated to ThiL inhibition. Together, these findings establish VP3.15 as the first validated small-molecule inhibitor of ThiL with antibacterial activity directly attributable to on-target inhibition.

### VP3.15 destabilizes ThiL *in vitro*

**Figure 6:**
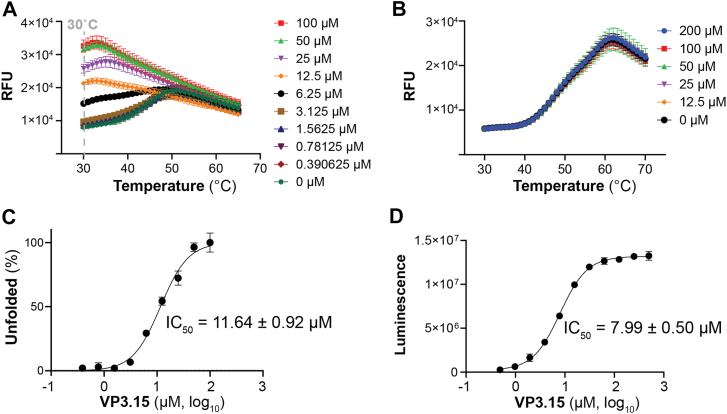
**Destabilization of ThiL by VP3.15.***A*, DSF of ThiL in the presence of varying concentrations of VP3.15. Protein unfolding was induced in a dose-dependent manner. *B*, DSF control run for VP3.15 using Falcilysin from *P. falciparum*. No protein unfolding was induced by VP3.15. *C*, comparison of ThiL unfolding at varying concentrations of VP3.15 at 30 °C. *D*, optimized enzymatic assay. Note the comparable IC_50_ values in panels *C* and *D*. Each data point represents the mean ± SD of triplicate. DSF, differential scanning fluorimetry; ThiL, thiamine monophosphate kinase.

**Figure 7:**
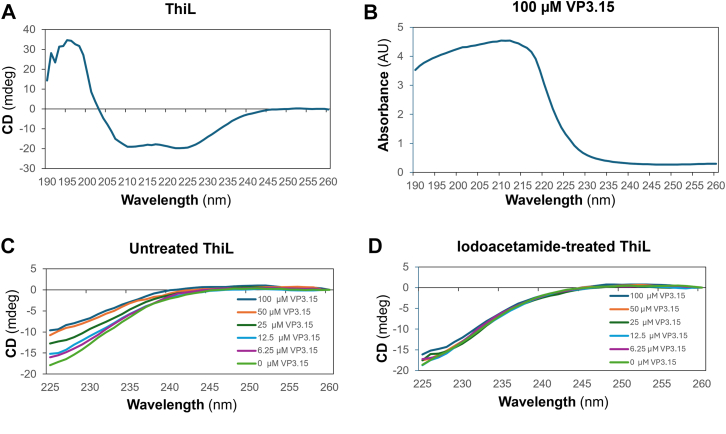
**Circular dichroism of ThiL protein.***A*, CD of ThiL in the absence of VP3.15. *B*, absorbance scan of VP3.15. At wavelengths shorter than 225 nm, absorbance was too high for reliable CD measurements. *C*, CD of ThiL in the presence of 0 μM to 100 μM VP3.15. Gradual unfolding of ThiL was induced by VP3.15. *D*, CD of iodoacetamide-treated ThiL in the presence of 0 μM to 100 μM VP3.15. Pretreatment with iodoacetamide protected ThiL from unfolding. ThiL, thiamine monophosphate kinase.

To characterize the mode of inhibition of VP3.15 and establish whether VP3.15 forms a stable complex with ThiL, we first performed isothermal titration calorimetry. Titration of VP3.15 into ThiL present in the vessel generated only background-level signals, comparable to the buffer-buffer negative control, whereas TMP (the ThiL ligand used as positive control) produced a clear release of heat corresponding to a dissociation constant Kd of 11.8 ± 3.72 μM (Fig. S3). Next, to better understand the interaction between VP3.15 and ThiL, we employed differential scanning fluorimetry (DSF), a biophysical technique that measures protein unfolding by tracking the fluorescence of the SYPRO orange hydrophobic dye. As ThiL is heated, it unfolds, exposing hydrophobic regions to the dye, provoking a significant increase in fluorescence (Fig. 6). This thermal shift assay relies on the enhancement of protein thermal stability upon binding of small-molecule ligands and is commonly used to screen for direct drug-target interactions. Surprisingly, using this assay, VP3.15 was found to induce unfolding of ThiL at a temperature of 30 °C prior to heating, in a concentration dependent manner (Fig. 6*A*). By contrast, VP3.15 did not induce unfolding of falcilysin from *Plasmodium falciparum*, (Fig. 6*B*) (19) nor of Zika virus RNA-dependent RNA polymerase (RdRp), two recombinant enzymes available in our laboratory that were used as control. ThiL unfolding by VP3.15 was concentration-dependent, with an apparent IC_50_ value of 11.64 ± 0.92 μM at 30 °C (Fig. 6*C*). This value correlates with the IC_50_ value of 7.99 ± 0.50 μM obtained in the optimized enzymatic assay (Fig. 6*D*; See Experimental procedure). The close agreement between the two IC_50_ values suggests that ThiL inhibition results from compound-induced destabilization of the enzyme. Consistently, the unfolding of ThiL was also observed using circular dichroism (CD) in which ThiL protein was measured in the presence of 0 μM to 100 μM VP3.15 (Fig. 7). In the absence of VP3.15, ThiL protein was well-folded (Fig. 7*A*), while the addition of VP3.15 caused ThiL to unfold gradually (Fig. 7*C*). Interestingly, when ThiL was preincubated with iodoacetamide, VP3.15 failed to induce the unfolding of ThiL (Fig. 7*D*), suggesting that free cysteines on ThiL (Fig. S4) might be important to VP3.15’s mechanism of action.

**Figure 8:**
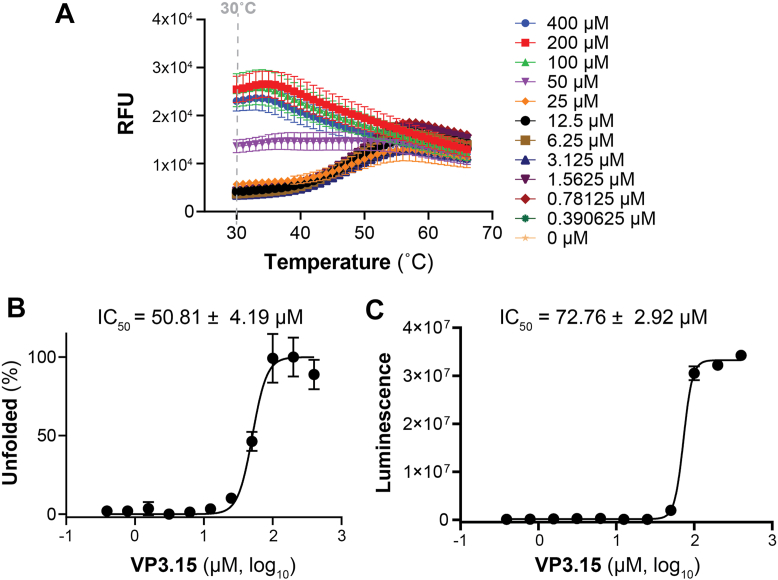
**Destabilization of GSK-3β by VP3.15.***A*, DSF of GSK3β in the presence of varying concentrations of VP3.15. Protein unfolding was induced in a dose-dependent manner. *B*, comparison of GSK-3β unfolding at varying concentrations of VP3.15 at 30 °C. *C*, GSK-3β kinase activity assay. Note the comparable IC_50_ values in *panelsB* and *C*. Each data point represents the mean ± SD of triplicate. DSF, differential scanning fluorimetry; GSK-3β, glycogen synthase kinase 3β.

As VP3.15 was originally developed as a glycogen synthase kinase 3β (GSK-3β) inhibitor for potential use to cure neuroinflammatory and neurodegenerative disorders (20), we next asked whether VP3.15 was also able to destabilize the structure of GSK-3β. Unfolding was also observed for purified recombinant GSK-3β when incubated with VP3.15 *in vitro* (Fig. 8) with an apparent IC_50_ value of 50.81 ± 4.19 μM at 30 °C. This observation suggests that the mechanism of the inhibitory activity of VP3.15 reported previously for GSK-3β may also involve protein destabilization. These findings raise the possibility that VP3.15 exerts broader effects on protein stability beyond ThiL, highlighting an additional dimension of its pharmacology that warrants further investigation. However, the observation that exogenous TPP rescued the antibacterial activity of VP3.15 in *P. aeruginosa* indicates that, in the bacterial context, ThiL is preferentially destabilized over other essential targets.

### Crystal structures of *Pa*ThiL and multimerization state

**Figure 9:**
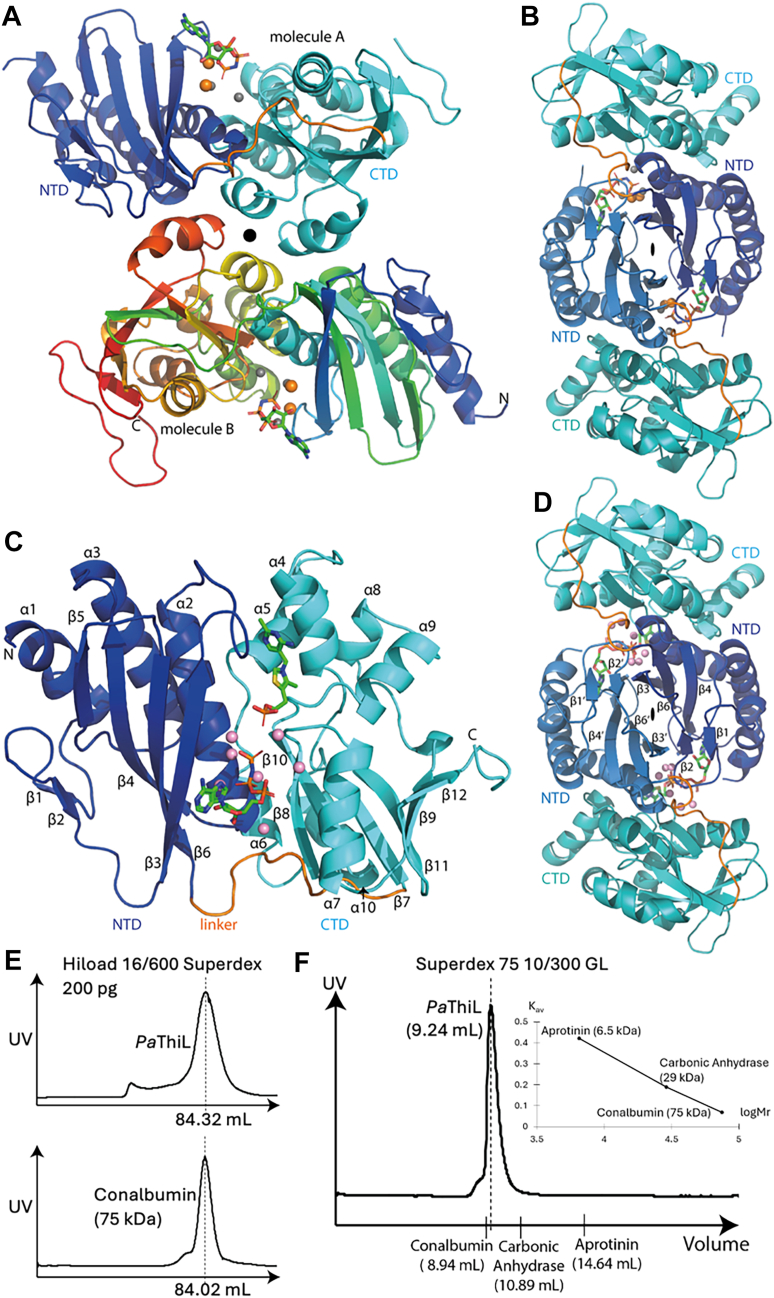
**Crystal structures of *Pa*ThiL, dimer formation, and location of the active site.***A*, binary complex of *Pa*ThiL and AMP-PNP coordinated by Mg^2+^ and Na^+^ ions. Two molecules of *Pa*ThiL are present in the asymmetric unit forming a noncrystallographic dimer related *via* a dyad (depicted by a *dot* •). For molecule A, the NTD is colored *blue* and CTD is colored *cyan*. The linker between NTD and CTD is colored *orange*. Molecule B is colored as “*rainbow*” from its N to C termini (cold to warm colors). Bound magnesium and sodium ions are shown as *gray* and *orange spheres*, respectively. The two AMP-PNP ligands are shown as *sticks* (*green*: carbon; *red*: oxygen; *blue*: nitrogen; *orange*: phosphorus). *B*, dimer A is formed by two copies of molecule A related by a two-fold crystallographic rotation symmetry (depicted by an *ellipse*). One protomer (*bottom*) is colored in *sky blue* (NTD) and *teal* (CTD); The other protomer (top) is colored in *blue* (NTD) and *cyan* (CTD). Dimer A represents a functional dimer with two equivalent active sites sitting in a cleft at the interface between the two protomers. *C*, ternary complex of *Pa*ThiL, AMP-PNP, and TMP coordinated by Ca^2+^ ions. Only one molecule of *Pa*ThiL is present in the asymmetric unit. The NTD is colored *blue* and CTD is colored *cyan*. The linker between NTD and CTD is colored *orange*. Bound calcium ions are shown as *pink spheres*. AMP-PNP and TMP are displayed as *sticks*. *D*, dimer C is formed by two copies of *Pa*ThiL from *panel C*, which are also related by a two-fold crystallographic rotation symmetry (depicted by an *ellipse*). *E*, SEC profiles of *Pa*ThiL (*upper panel*) and conalbumin (*lower panel*) in Hiload 16/600 Superdex 200 pg column. Both proteins share a similar elution volume, suggesting a comparable particle size. *F*, SEC profile of *Pa*ThiL in Superdex 75 10/300 Gl column. Calibration of the column was performed with three proteins (conalbumin, carbonic anhydrase, and aprotinin), and their elution volumes are marked on *x-axis*. The linear relationship between K_av_ and logM_r_ allows estimation of the molecular weight of the *Pa*ThiL peak, which is approximately 65 kDa, suggesting a dimer formation. AMP-PNP, adenylyl-imidodiphosphate; CTD, C-terminal domain; NTD, N-terminal domain; PaThiL, pseudomonas aeruginosa thiamine monophosphate kinase; TMP, thiamine monophosphate.

Given the potential of ThiL as an antibacterial target as substantiated above, two high-resolution crystal structures were obtained (Table S1): a binary complex at a resolution of 1.8 Å of *Pa*ThiL bound with adenylyl-imidodiphosphate (AMP-PNP) coordinated by Mg^2+^ and Na^+^ (Fig. 9, *A* and *B*) and a ternary complex at a resolution of 2.17 Å of *Pa*ThiL bound with AMP-PNP, TMP, and Ca^2+^ (Fig. 9, *C* and *D*). The *Pa*ThiL monomer contains seven solvent-exposed free cysteine residues evenly scattered across both its two N- and C-structural domains (Figs. S4 and S6). The binary complex (space group C222_1_) contains two molecules of *Pa*ThiL arranged as a dimer in the asymmetric unit, with both monomers sitting around a noncrystallographic 2-fold axis (Fig. 9*A*). However, the surface area buried of only 547 Å^2^ indicates that this dimer (Fig. 9*A*) is provoked by crystal packing forces. Instead, both molecule A and B form a 2-fold symmetric homodimer that share a very similar arrangement with a r.m.s.d. of 0.59 Å between 564 superimposed Cα atoms (Fig. 9*B*). The surface area buried between the two protomers is 1734 Å^2^ for dimer A and 1714 Å^2^ for dimer B. The ternary complex structure determined in space group P6_1_22, contains one molecule of *Pa*ThiL per asymmetric unit (Fig. 9*C*). The same 2-fold symmetric homodimer -dimer “C”- is also formed (Fig. 9, *B* and *D*) with a r.m.s.d. of 1.61 Å over 586 Cα atoms compared to dimer A, and the individual protomers overlap closely with a r.m.s.d. of 0.35 Å for 234 Cα atoms. To determine the multimeric state of *Pa*ThiL in solution, we performed size-exclusion chromatography (Fig. 9*E*). Conalbumin, a monomeric globular protein with an Mr of 75 kDa, used as standard, eluted at 84.02 ml. *Pa*ThiL (Mr of 35 kDa) eluted at 84.32 ml, showing that *Pa*ThiL forms a stable dimer in solution (Fig. 9*F*).

Like its bacterial homologs, the *Pa*ThiL protomer consists of two domains: an N-terminal domain (NTD) spanning Met1 to Pro139 and a C-terminal domain (CTD) from Asp153 to Asp322. The NTD and CTD are connected by a 13-residues-long linker spanning Ala140 to Gly152 (Fig. 9*C*). The NTD domain adopts an α/β-fold with connectivity: α1, β1, β2, β3, α2, β4, α3, β5, and β6. A twisted β-sheet is formed by strands β3↓β6↑β4↓β5↓, flanked by helices α1, α2, and α3 on one side, with two additional short β strands (β1 and β2) sitting next to strand β5. The CTD also adopts an α/β fold contributed by β7, α4, α5, α6, β8, α7, β9, α8, α9, β10, α10, β11, and β12. A twisted β-sheet is formed by β8↓β10↑β7↓β11↑β9↓β12↓ flanked by helices α6 and α10 on one side of the β-sheet and helix α7 on the other side. Besides the linker connecting NTD to CTD, these domains interact through helices α2, α5, α6, and the loop between helices α5 and α6.

The *Pa*ThiL dimer is mainly stabilized *via* interactions formed between the twisted β-sheets β3↓β6↑β4↓β5↓ of both NTDs (Fig. 9*D*). Interestingly, strands β1 and β2 also contribute to dimerization by extending the twisted β-sheet β3↓β6↑β4↓β5↓ of the other protomer, resulting in a continuous intermolecular β-sheet β1’↓β2’↑β3↓β6↑β4↓β5↓, where prime strands denote strands from the monomer related by the 2-fold crystallographic symmetry.

An automated structural homology search (21) was performed using the *Pa*ThiL orthorhombic crystal structure (this work) (Table S2). The closest structures identified are ThiL from *Acinetobacter baumannii* (*Ab*ThiL), *Stenotrophonomas maltophilia* (*Sm*ThiL), *Methilobacillus flagellatus* (*Mf*ThiL), *Aquifex aelolicus* (*Aa*ThiL) and *Thermus thermophilus* (*Tt*ThiL). These ThiL homologs all form 2-fold symmetric homodimers and share similar structures, despite relatively low amino-acid sequence identities ranging from 31 to 45%. Based on the different folds adopted by their N-terminal regions, bacterial ThiL structures can be partitioned into two groups (Fig. S5): The first group consists of *Pa*ThiL, *Ab*ThiL, *Sm*ThiL, and *Mf*ThiL, whose N-terminal region is adjacent to helix α3 from the same protomer. The second group consists of *Aa*ThiL and *Tt*ThiL, whose N-terminal region is adjacent to helix α3′ from the other protomer. In this group, the β1 strand from the N-terminal region contributes to the intramolecular β-sheet β1↑β2↓β5↑β3↓β4↓ *via* domain exchange (Fig. S6). The metal-coordinating residues Asp30, Asp47, Asp75, and Asp211 have been strictly conserved during evolution. Other evolutionary-conserved residues are found in two loops near the active site comprising: Gly28, Asp29 and Asp30 that connect strands β1 and β2, and residues Ser213, Asp214, Gly215, and Leu216 between strand β8 and helix α7.

Two equivalent active sites are located in the cleft housed at the dimer interface with AMP-PNP coordinated by one protomer, TMP coordinated by the other protomer, and their phosphate tails stabilized by metal ions (Fig. 9, *B* and *D*). In both the binary and ternary complexes of *Pa*ThiL, coordination of AMP-PNP is conserved: the adenine base inserts in a hydrophobic pocket shaped by helix α1 and strands β5 and β4 (Fig. S7, *A* and *B*), while hydrogen bonds are formed between the side chain of Thr123 and the N6 atom of the adenine base and between Thr123 and the N7 atom (Fig. S7, *C* and *D*). The backbone amine groups of Gly28 and Gly121 form hydrogen bonds with O3 and O2 of the ribose moiety, respectively. However, the AMP-PNP phosphate tails adopt two distinct conformations at the α- and β-phosphates. This structural change is probably attributable to the different metal ions employed for charge stabilization.

### The binary complex of *Pa*ThiL bound with AMP-PNP coordinated by Mg^2+^ and Na^+^

Here, three Mg^2+^ and two Na^+^ ions present at the active site are all coordinated octahedrally. The first Mg^2+^ ion (Mg_1_^2+^) is coordinated by the α, β, γ-phosphates of AMP-PNP, Asp122 and two water molecules (Fig. S7*C*). Asp122 is conserved in the ThiL homologs except in *Aa*ThiL in which an asparagine residue is used instead for Mg^2+^ coordination. The second Mg^2+^ ion (Mg_2_^2+^) is coordinated by the γ-phosphate of AMP-PNP, two water molecules and the strictly conserved residues Asp75′ and Asp47′ from the other protomer. A third Mg^2+^ ion (Mg_3_^2+^) is coordinated by Asp30, three water molecules and residues Asp75′ and Asp211′ from the other protomer (Fig. S6). The first Na^+^ ion (Na4^+^) is coordinated by the γ-phosphate of AMP-PNP, Asp122, two water molecules, and Asp47′ while the second Na^+^ ion (Na5^+^) by Asp30, three water molecules, Asp75′ and Ser45′ (Fig. S7*C*).

### Ternary complex of *Pa*ThiL bound with AMP-PNP, TMP, and Ca^2+^

Instead of the Mg^2+^ and Na^+^ ions, seven Ca^2+^ ions are found at the active site (Fig. S7*D*), due to the presence of 0.2 M calcium acetate in the crystallization buffer. Four Ca^2+^ ions are located at the positions occupied by Mg_2_^2+^, Mg_3_^2+^, Na_4_^+^, and Na_5_^+^ in the binary complex. Ca_2_^2+^ of the ternary structure replaces Mg_2_^2+^ as the cation bridges Asp75′, Asp47′ and the γ-phosphate of AMP-PNP, while Ca_4_^2+^ replaces Na_4_^+^ as the cation bridging Asp122, Asp47′, and the γ-phosphate of AMP-PNP. Although Ca_3_^2+^ shares a similar position as Mg_3_^2+^ and is also coordinated by Asp30, Asp75′ and Asp211′, it establishes interactions with the β and γ-phosphates. As a result, the phosphate tail is pulled toward the calcium ion. Similarly, Ca_5_^2+^ and Na_5_^+^ share coordinating residues Asp30, Asp75′ and Ser4 but additional ionic interactions are formed between Ca5^2+^ and AMP-PNP β-phosphate group, pulling it toward the cation. These additional ionic interactions stem from the flexibility of the AMP-PNP phosphate tail and the charge and coordination distances of Ca^2+^ (Fig. S7*D*). In contrast, in the presence of Mg^2+^ and Na^+^ which differ from Ca^2+^ in both charge and coordination distance, these additional ionic interactions could not be established and the AMP-PNP phosphate tail adopts another conformation (Fig. S7*C*). The other three Ca^2+^ ions adopt positions unique to the ternary structure. Two (Ca6^2+^ and Ca7^2+^) stabilize active site residues Asp29, Asp30, Asp214′, Asp219′, and His222′ . Finally, Ca1^2+^ bridges Asp214′ and the phosphate tail of TMP, while TMP makes hydrogen bonds with the side chains of His54′ and Tyr189′ (Fig. S7*D*).

The conversion of ATP and TMP to ADP and TPP by ThiL involves the transfer of ATP γ-phosphate to TMP through an in-line attack of TMP α-phosphate on ATP γ-phosphate (22, 23). This reaction mechanism requires close proximity of the ATP γ-phosphate and TMP α-phosphate. This scheme is supported by crystal structures of both *Ab*ThiL ternary complexes and *Aa*ThiL ternary complexes. For *Ab*ThiL (PDB accession code: 5DD7) and *Aa*ThiL (PDB accession code: 3C9T), the distance between the AMP-PNP γ-phosphate and TMP α-phosphate is 3.3 Å (Fig. S7, *E* and *F*). Interestingly, in the *Pa*ThiL ternary structure obtained here, which employs Ca^2+^ as the metal cofactor, the AMP-PNP γ-phosphate and TMP α-phosphate are separated by 5.3 Å (Fig. S7*D*). This distance is incompatible with an in-line nucleophilic attack suggesting that the *Pa*ThiL ternary structure represents an inactive complex. Consistently, *Pa*ThiL displayed no kinase activity with calcium ions as the cofactor (Fig. S8).

## Discussion

This study demonstrates that *P. aeruginosa* relies on *de novo* TPP biosynthesis to sustain growth and cause infection, establishing ThiL, the enzyme catalyzing the final step in TPP synthesis, as an essential metabolic vulnerability. Although vitamin biosynthetic pathways have long been considered attractive antibacterial targets, their relevance has often been questioned due to the presumed ability of bacteria to salvage thiamine derivatives from the host environment (11). Our findings combined with prior work (16) challenge this assumption by showing that *P. aeruginosa* cannot efficiently import TPP and therefore cannot bypass ThiL inhibition. Indeed, growth of the *P. aeruginosa* Δ*thiL* strain was only possible in the presence of supraphysiological concentrations of TPP (∼5 mM), far exceeding levels reported in host tissues (17). This strict dependency directly impacts the capacity of the pathogen to replicate and disseminate during infection, positioning ThiL as an essential enzyme during infection.

A recurring concern for vitamin-pathway targets is whether host-derived thiamine derivatives can bypass inhibition during infection. Our data argue that this is unlikely for *P. aeruginosa*: exogenous TPP only rescued growth at supraphysiological concentrations, and Δ*thiL* viability required millimolar supplementation, indicating that intracellular TPP synthesis remains effectively indispensable in host-relevant settings. This mitigates a key translational liability for ThiL inhibition, and supports its prioritization as an infection-relevant vulnerable target.

Importantly, we provide further *in vivo* demonstration that disruption of TPP metabolism profoundly attenuates *P. aeruginosa*. Our work expands on earlier findings showing that disruption of TPP metabolism attenuates *P. aeruginosa* virulence in the lung of immunocompetent mice (16). By employing a broader set of infection models, including skin, and pulmonary infection, we demonstrated that the *thiL* KO strain exhibits drastically reduced replication and an inability to disseminate to secondary tissues. These results consolidate and extend the concept that thiamine metabolism is indispensable for *P. aeruginosa* pathogenesis across multiple host niches. Although metabolic enzymes have historically been underexplored in antibiotic discovery compared to classical bactericidal targets, our data strengthen the case for revisiting bacterial metabolism as a reservoir of therapeutic opportunities (5). Leveraging on this insight, we developed an enzymatic assay to identify small-molecule inhibitors of ThiL and report VP3.15 as the first compound to selectively inhibit *P. aeruginosa* ThiL with validated cellular activity. Unlike previously described inhibitors (16), the antibacterial effect of VP3.15 was fully reversible by exogenous TPP supplementation, confirming its on-target mechanism.

Biophysical assays using isothermal titration calorimetry and also direct attempts to cocrystallize VP3.15 with ThiL failed to demonstrate direct binding of VP3.15 to ThiL. Instead, thermal shift assays revealed that the compound promotes destabilization and unfolding of the enzyme *in vitro*. This suggests that VP3.15 does not act through classical competitive inhibition but instead perturbs protein stability. Such a mechanism is rare among antibacterial compounds, making these findings novel but mechanistically intriguing. Although protein destabilization is more commonly associated with targeted protein degraders in eukaryotic systems (24), our data raise the possibility that small molecules could exploit similar strategies in bacteria, albeit further work is needed to fully understand the mechanism. The selective antibacterial rescue of VP3.15 by TPP argue against a purely artefactual effect. Instead, our findings align more closely with the emerging concept of protein-destabilizing compounds, which directly perturb folding and promote selective loss of function in their targets (25). Such a mechanism, while still uncommon in antibacterial discovery, highlights the potential of exploiting protein stability as an alternative antibacterial modality in bacteria.

The ThiL structures were initially solved with the objective of obtaining a costructure with VP3.15 to support a structure-based interpretation of inhibition. However, once VP3.15 was found to destabilize ThiL rather than behave as a classical ligand forming a stable complex, we did not pursue this further. Nevertheless, the structures remain valuable as they define the active-site architecture and establish a framework for future structure-guided discovery of ThiL inhibitors.

The broader pharmacological implications of VP3.15 are noteworthy. The compound VP3.15 was originally developed as a GSK-3β inhibitor for neurodegenerative disorders (20). Consistent with its unusual mode of action against ThiL, we further observed that VP3.15 destabilizes GSK-3β *in vitro*, suggesting that protein destabilization may underlie its activity across distinct targets. Such a mechanism has not previously been reported for GSK-3β inhibitors and could represent an underappreciated aspect of its potency. The fact that VP3.15 destabilizes multiple proteins but not all those tested (such as the parasite Zinc protease falcilysin or the viral Zika virus RdRp) further highlights that its effects on protein stability may be selective, albeit not limited to ThiL. This duality, preferential targeting of ThiL in the cytoplasm of *P. aeruginosa*, alongside destabilization of mammalian GSK-3β, illustrates both the promise and the challenges of repurposing or adapting such scaffolds for antibacterial discovery.

Although these findings support a nonclassical inhibitory mechanism, it is important to consider the potential chemical liabilities of VP3.15. VP3.15 was evaluated using the PAINS filters implemented in SwissADME, which did not flag any structural alerts. However, we acknowledge that such *in silico* filters are not exhaustive and may not capture all classes of reactive or covalent modifiers. For example, several classes of compounds, including isothiazolones, hydroxyphenyl hydrazones, and ene-rhodanine, are capable of covalently modifying proteins (26). Although VP3.15 does not contain any chemical moieties associated with known PAINS, our circular dichroism results suggest that free cysteines play an important role in its mechanism of action. Specifically, a high density of solvent exposed free cysteines is a shared feature of ThiL and GSK-3β, with ThiL containing seven cysteines (2.2% of total residues) and GSK-3β containing nine (2.1%) (Fig. S4). In contrast, Zika RdRp contains 9 cysteines which are less solvent exposed, while falcilysin harbors only 3 cysteines, representing just 0.3% of its total residues. Whether VP3.15 engages ThiL through covalent modification or an alternative cysteine-dependent mechanism remains to be determined and could be addressed in future studies.

The identification of VP3.15 underscores the need for broader screening campaigns to discover alternative chemical scaffolds that inhibit ThiL through more conventional or higher-affinity mechanisms.

More generally, our work highlights the opportunity of targeting bacterial cofactor biosynthesis pathways. Similar approaches have been explored for folate (27, 28), menaquinone (8), and NAD biosynthesis (29, 30), often with promising results in both biochemical and infection models. Thiamine metabolism adds to this growing list and presents the advantage of being indispensable in certain pathogens under host conditions. However, one limitation is that the degree of dependency on *de novo* thiamine synthesis and the presence of salvage pathways may vary among bacteria. Some pathogens retain efficient salvage pathways or dedicated transporters that may reduce susceptibility to ThiL inhibition (31). It will therefore be essential to expand comparative studies across gram-negative species to assess the generalizability of this vulnerability. In conclusion, this study establishes ThiL as an essential and druggable metabolic vulnerability in *P. aeruginosa*, validates a pharmacological strategy to exploit this enzyme, and introduces a unique mechanism of inhibition based on protein destabilization. By integrating genetic, biochemical, structural and infection model data, we provide a comprehensive case for ThiL as a high-value antibacterial target. Although significant work remains to discover more potent inhibitors and to define the broader applicability of this approach, our findings open the door to targeting bacterial metabolism as a means of overcoming multidrug resistance. This framework may serve as a template for exploring other metabolic dependencies in clinically relevant gram-negative pathogens.

## Experimental procedures

### Bacterial strains, culture media, and chemicals

Bacteria strains were maintained in Difco Luria-Bertani (LB10) broth or agar (Miller) and M9 minimal medium (Sigma-Aldrich). The M9 medium contained 48 mM Na_2_HPO_4_, 22 mM KH_2_PO_4_, 9 mM NaCl, and 19 mM NH_4_Cl, supplemented with 20 mM D-glucose, 2 mM MgSO_4_, 0.2% (w/v) casamino acids, and trace elements. Bacteria were cultured in M9 minimal medium at 37 °C with shaking at 200 rpm. Δ*thiC*, Δ*thiE*, and Δ*thiG* KO mutant were cultured in minimal media supplemented with 40 nM thiamine and Δ*thiL* KO mutant was cultured in minimal media supplemented with 5 mM TPP for normal growth.

### Minimum inhibitory concentration determination

Bacteria were cultured overnight in M9 minimal medium at 37 °C with shaking. Overnight cultures (OD600 = 0.01) were inoculated into fresh medium and grown to mid-log phase (OD600 = 0.2–0.5). Cells were harvested by centrifugation at 3800 rpm for 10 min, washed twice, and resuspended in M9 minimal medium. The suspension was adjusted to a final OD600 of 0.001. Test drugs or thiamine derivatives were serially diluted (1:2) in M9 medium and mixed with the bacterial suspension at a 1:1 ratio in 96-well plates. Plates were incubated at 37 °C for 16 to 20 h, and OD600 was measured using a Cytation 3 plate reader (BioTek). Data were analyzed using nonlinear regression and fitted to a sigmoidal dose-response curve in Prism software.

### Murine infection models

Female Balb/c mice (6–8 weeks old) were obtained from *InVivos* and quarantined for one week before experimentation. Bacteria were cultured overnight in M9 minimal medium at 37 °C with shaking. Subcultures were inoculated (OD600 = 0.01) into fresh medium and grown to mid-log phase (OD600 = 0.2–0.5). Bacterial cells were harvested by centrifugation at 3800 rpm for 10 min, washed twice, and resuspended in M9 minimal medium. The OD600 was measured, and suspensions were adjusted to a final concentration of 10^4^–10^5^ colony-forming units (CFU) in 5 μl of PBS. Mice were weighed and anesthetized *via* intraperitoneal injection of a ketamine (100 mg/kg) and xylazine (10 mg/kg) cocktail. The dorsal fur was shaved, disinfected with 70% ethanol, and a circular wound (∼0.5 cm radius) was created. A 5 μl bacterial suspension (10^4^–10^5^ CFU) was inoculated into the wound, which was then covered with a sterile dressing. Tramadol (200 μl per mouse) was administered intraperitoneally for pain management. After 24 h, mice were euthanized using carbon dioxide, followed by decapitation or cervical dislocation. A 1 cm × 1 cm section of infected skin was excised for analysis. For the lung infection model, mice received intraperitoneal cyclophosphamide (150 mg/kg) fourdays before infection and 100 mg/kg one day before infection to induce immunosuppression. A 50 μl bacterial suspension (∼10^6^ CFU) was administered intranasally. After 15 h, mice were sacrificed, and lungs, livers, and spleens were harvested. Excised tissues were placed in 1 ml PBS within a soft tissue homogenization tube containing ceramic beads (Bertin Instruments). Tissue homogenization was performed using a Precellys 24 homogenizer (Bertin Instruments) at 4600 rpm for 2 × 10 s, with a 10-s pause between cycles. Serial dilutions were plated on LB agar, and CFU were enumerated. All experiments were conducted in accordance with institutional guidelines and approved by the Institutional Animal Care and Use Committee (IACUC) of Nanyang Technological University, Singapore (IACUC protocol A19100 and A24055).

### Cloning, expression, and purification

For ThiL, the gene coding for ThiL (WP_003113052.1) was amplified from genomic DNA and cloned into plasmid pNIC28-Bsa4 downstream of a 6 × His affinity tag and a TEV cleavage site. The plasmid was transformed into BL21 (DE3) Rosetta T1R and bacteria cultured in LB media with kanamycin and chloramphenicol at 37 °C until the OD_600_ reached 0.6 to 0.8. Subsequently, the temperature was decreased to 16 °C and 0.5 mM Isopropyl β-D-1-thiogalactopyranoside (Affymetrix) was added to the bacterial culture for overnight protein expression. Bacteria were harvested the next day by centrifugation at 4000*g* for 15 min and resuspended in lysis buffer (20 mM Na Hepes, 500 mM NaCl, 10% (v/v) glycerol, and 0.5 mM Tris (2-carboxyethyl) phosphine (TCEP) at pH 7.5). Cells were either stored at −80 °C or lysed immediately using a LM20 microfluidizer. The cell lysate was centrifuged at 48,000*g* for 1 h at 4 °C and the supernatant was incubated with 2 ml Ni-NTA resin for 1 h before loading into a gravity column. Subsequently, the Ni-NTA resin was washed stepwise with 50 ml of lysis buffer, with 10 ml of wash buffer 1 (lysis buffer with an additional 10 mM imidazole) and finally with 10 ml of wash buffer 2 (lysis buffer with 20 mM imidazole). The ThiL protein was eluted from the resin with 9 ml of elution buffer (lysis buffer supplemented with 1 M imidazole) and collected as 1 ml fractions whose content were subsequently checked by sodium dodecyl sulfate polyacrylamide gel electrophoresis. The fractions containing eluted ThiL protein were pooled and loaded onto a Hiload 16/600 Superdex 200 pg size-exclusion chromatographic column (GE Healthcare) preequilibrated in buffer (20 mM Na Hepes, 300 mM NaCl, 10% (v/v) glycerol, and 0.5 mM TCEP at pH 7.5). Protein purity was confirmed by sodium dodecyl sulfate polyacrylamide gel electrophoresis and ThiL was concentrated to 61.5 mg/ml, flash-frozen in liquid nitrogen, and stored at −80 °C until use.

### Screening inhibitor library

A library of 1231 kinase inhibitors (MedChemExpress) was screened to identify potential inhibitors of ThiL protein activity. ThiL kinase function was evaluated by quantifying ATP depletion using a luminescence-based assay. The assay was conducted in a 25 μl reaction mixture containing 50 mM Tris-HCl (pH 7.0), 50 μM TMP, 10 μM ATP, 5 mM MgCl_2_, 350 mM KCl, 1 μM ThiL protein, and 10 μM test compound (14). Reactions were incubated at 37 °C for 30 min. Following incubation, an equal volume of CellTiter-Glo reagent (Promega) was added to each well. The mixture was further incubated at room temperature for 10 min to stabilize the luminescent signal, which was then measured using a Cytation 3 plate reader (BioTek).

### Optimized enzymatic assays for inhibitor VP3.15

ThiL enzymatic activity was measured using the Kinase-Glo Plus Luminescent Kinase Assay Kit (Promega). Reactions were performed in buffer containing 50 mM Tris-HCl, pH 7.5, 5 mM MgCl_2_, and 350 mM KCl. Each reaction contained 5 μM of purified ThiL, 25 μM ATP, 200 μM TMP, and varying concentrations of VP3.15. Samples were incubated at 37 °C for 20 min, after which 2 mM TCEP was added. Kinase-Glo Assay Buffer was then introduced, and the mixtures were incubated at room temperature for 10 min to stabilize the luminescent signal. Luminescence was recorded using a Spark Multimode Microplate Reader (TECAN Life Sciences) with an integration time of 1 s per well. GSK3β kinase activity was assessed under similar conditions. Each reaction contained 5 μM of purified GSK3ß, 25 μM ATP, 25 μM GS-2 peptide substrate (MedChemExpress), and varying concentrations of compounds (200 μM to 390.625 nM).

### DSF of proteins with compounds VP3.15

DSF experiments were carried out using the CFX96 Touch Real-Time PCR Detection System (Bio-Rad) and in nonreducing buffer (20 mM Na Hepes, 300 mM NaCl, and 10% (v/v) glycerol at pH 7.5). Each 25 μl reaction contained 5 μM of purified protein, 5X SYPRO orange dye and varying concentrations of compounds (200 μM to 390.625 nM). Melt curves were obtained by heating the plate from 30 to 95 °C with a heating rate of 1 °C/10 s. RFU was measured using the FRET channel. The IC_50_ values of protein unfolding induced by VP3.15 were estimated by comparing RFU values at the 30 °C cross section.

### Circular dichroism spectroscopy

Far-UV circular dichroism spectra were recorded using an Chirascan Circular Dichroism Spectrometer (Applied Photophysics) with a 1 mm pathlength quartz cuvette. ThiL protein was diluted to 0.2 mg/ml in 20 mM Na Hepes, 300 mM NaCl, and 10% (v/v) glycerol at pH 7.5. CD spectra were collected in the presence of 0 to 100 μM VP3.15 at room temperature. Initial scans were collected from 190 to 260 nm; however, due to high absorbance of the VP3.15 stock, the wavelength range was restricted to 225 to 260 nm to maintain acceptable signal quality. Spectra were acquired with a 1.5 s integration time per point, 1 nm step size, and 1 nm bandwidth. For iodoacetamide pretreatment, ThiL protein was incubated with 10 mM iodoacetamide for 3 h on ice, followed by overnight dialysis to remove excess iodoacetamide. The treated protein was then diluted to 0.2 mg/ml and analyzed by CD under the same conditions as the untreated sample.

### Crystallization, data collection, structure determination, refinement, and analysis

To obtain the *Pa*ThiL binary complex, a mixture of 30 mg mL^−1^ PaThiL, 5 mM AMP-PNP, and 5 mM MgCl_2_ was screened against various commercial crystallization kits including Morpheus and JCSG-plus (Molecular Dimensions), Index and PEG/Ion Screen (Hampton Research) using a mosquito crystallization robot (TTP Labtech). Cocrystals were obtained in Morpheus H9 condition: 0.02 M DL-glutamic acid monohydrate, 0.02 M DL-alanine, 0.02 M glycine, 0.02 M DL-lysine monohydrochloride, 0.02 M DL-serine, 0.061 M Tris HCl, 0.039 M Bicine, pH 8.5, 20% v/v PEG 500 MME, and 10% w/v PEG 20000. These crystals were flash-frozen in liquid nitrogen and shipped to Australian Synchrotron beamline MX1 for remote data collection. Diffraction images were processed using XDS (32), and the structure was determined with the molecular replacement program MolRep (33) using the *Sm*ThiL crystal structure (PDB access code: 6XEP) as a search probe. Manual building using COOT (34) was combined with refinement using Phenix Refine (35). Two ThiL molecules are present in the asymmetric unit but do not form a stable dimer. Quality of the structure was improved using rotamer outlier, Ramachandran outlier and geometric constraints. Metal ions (Mg^2+^ and Na^+^) were built based on coordination distances and electron density strength, and confirmed using CheckMyMetal (36).

To obtain the *Pa*ThiL ternary complex, a mixture of 30 mg mL^−1^ *Pa*ThiL, 5 mM AMP-PNP, 5 mM MgCl_2_, and 5 mM TMP was also screened against the above-mentioned crystallization kits. Cocrystals were obtained in JCSG-plus D10: 0.2 M calcium acetate hydrate, 0.1 M sodium cacodylate, pH 6.5, and 40% (v/v) PEG 300. The subsequent steps were performed in the same way as the binary complex crystal.

### Estimation of *Pa*ThiL size in solution

The void volume (V_0_) of the Superdex 75 10/300 Gl column was first determined through injection of Blue Dextran 2000. Next, three protein standards (conalbumin, carbonic anhydrase, and aprotinin) as well as *Pa*ThiL were loaded onto the column to measure their respective elution volumes (V_e_). The partition coefficient K_av_ for each protein was calculated using the equation $K_{av}=\frac{V_{e}-V_{0}}{V_{C}-V_{0}}$, where V_c_ represents the geometric column volume. A calibration curve was constructed by plotting the K_av_ values of the standards against the logarithms of their molecular weights (logM_r_). Using the calibration curve, logM_r_ of *Pa*ThiL was estimated based on the linear relationship between K_av_ and logM_r_. All size-exclusion chromatography runs were performed in the same buffer.

## Data availability

Atomic coordinates and structure factors for the ThiL crystal structures reported here have been deposited to the PDB (www.rcsb.org) with access codes 8YKS (binary complex between *Pa*ThiL and AMPPNP) and 8YKU (ternary complex between *Pa*ThiL with AMPPNP and TMP).

## Supporting information

This article contains supporting information.

## Conflict of interest

The authors declare that they have no conflicts of interest with the contents of this article.
