# Supplementary material for: A thiadiazolylidene-morpholine compound inhibits Pseudomonas aeruginosa by destabilizing the thiamine monophosphate kinase thiL
Source: J Biol Chem. 2026 May 6;302(6):113112. doi: 10.1016/j.jbc.2026.113112 (PMC13254938; doi:10.1016/j.jbc.2026.113112)
Supplement: Supporting Information [file mmc1.docx]

A thiadiazolylidene-morpholine compound inhibits *Pseudomonas aeruginosa* by destabilising the thiamine monophosphate kinase thiL

Yingying Li^1#^, Jianqing Lin^2,3#^, Zara Shi Ying Chung ^2,3^, Benny Ken Yee Yeo ^1^, Julien Lescar^2,3 4,*^, Kevin Pethe^1,4,5,6,*^

Contents

Supplementary Figures

Figure S1. Bacterial loads in mice lungs infected with PAO1, Δ*thiL*, or Δ*thiL* complement.

Figure S2. Purification of recombinant ThiL and activity in DMSO

Figure S3. Isothermal Titration Calorimetry showed no detectable binding between VP3.15 and ThiL.

Figure S4. Free cysteine residues of (A) ThiL and (B) GSK3ß.

Figure S5. Different orientations of the N-terminal regions.

Figure S6. Structure-based sequence alignment of ThiL homologues.

Figure S7. Close-up views of the *Pa*ThiL active site.

Figure S8. Metal dependency of *Pa*ThiL kinase activity.

Supplementary Tables

Table S1. Data collection and structure refinement statistics of *Pa*ThiL crystals.

Table S2. Summary of DALI structural homology search results for *Pa*ThiL.

**
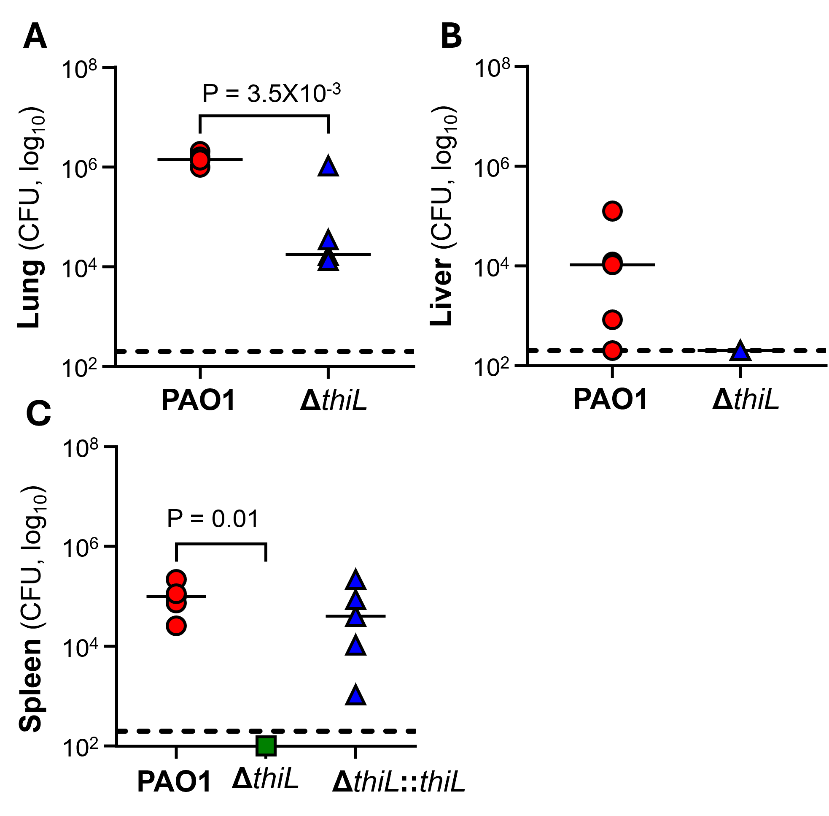
Supplementary Figure S1.** ThiL is required for virulence in mice. (A-B) Immunocompetent mice were infected by the PAO1 or PAO1Δ*thiL* strains by the intranasal route. Bacterial burden was determined in the lung (A) and liver (B) 15 hours post-infection by CFU determination on agar plates. (C) Neutropenic mice were infected by the PAO1, PAO1Δ*thiL*, or PAO1Δ*thiL*::*thiL* strains by the intranasal route. Bacterial burden was determined in the spleen 15 hours post-infection. Same group of mice reported Fig. 2A and Fig 2B. Data represent mean ± SD of five mice per group. Statistical analysis was performed using one-way ANOVA followed by Tukey’s multiple comparisons test.

**
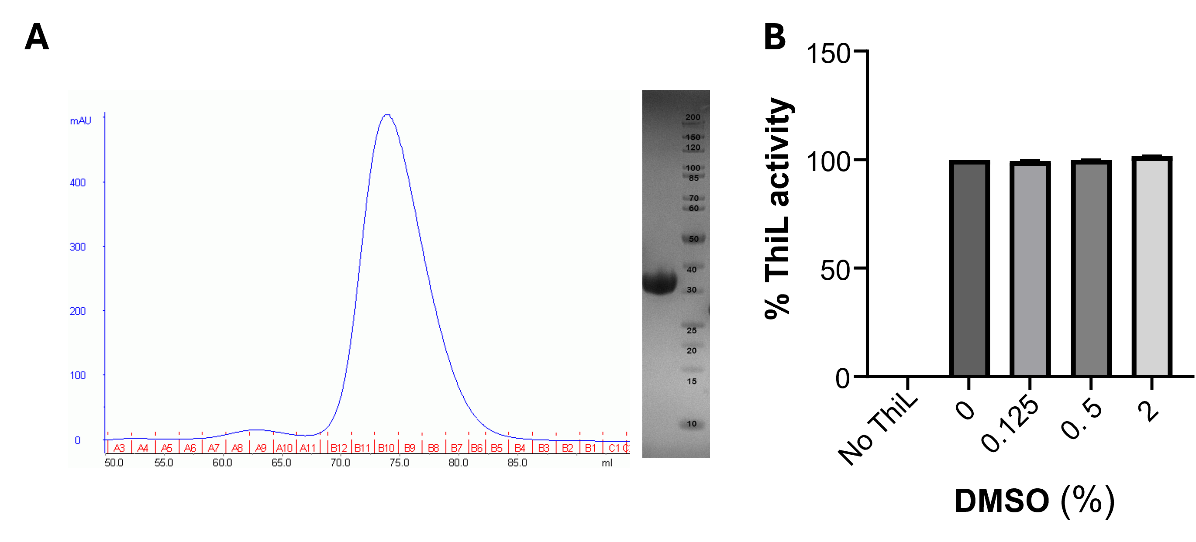
Supplementary Figure S2.** Purification of recombinant ThiL. (A) Recombinant purification of ThiL protein. Left: Size-exclusion chromatography in Hiload 16/600 Superdex 200 pg. Right: Final protein purity analysed by SDS-PAGE. (B) ThiL enzymatic activity is not impacted significantly by DMSO up to a concentration of 2 %.

**
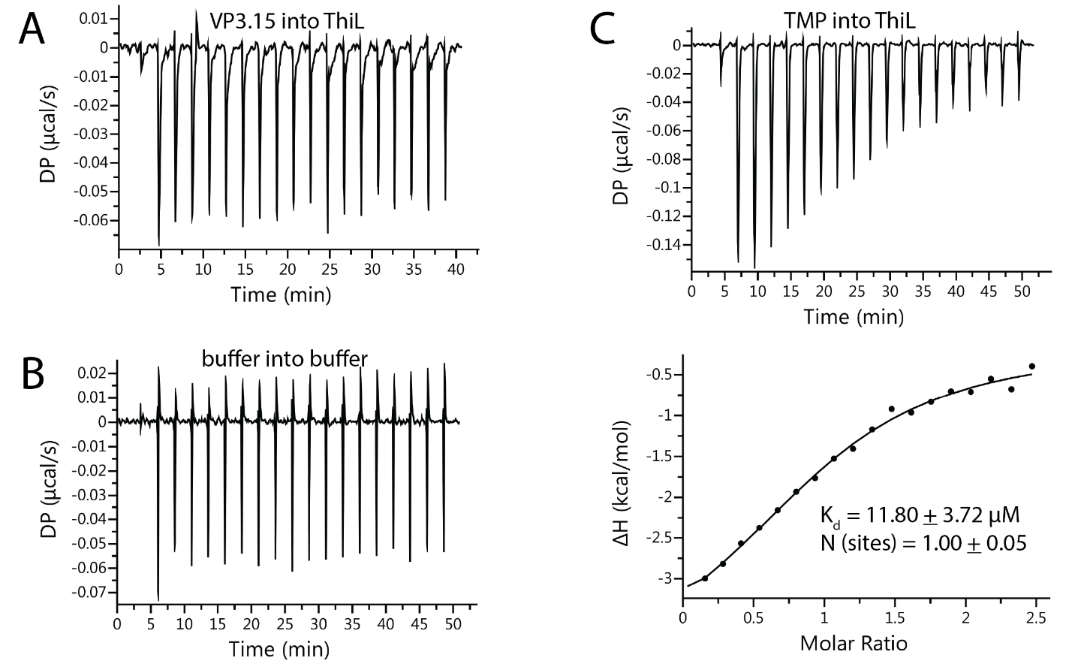
Supplementary Figure S3.** Isothermal Titration Calorimetry showed no detectable binding between VP3.15 and ThiL. (A) 30 µM of ThiL was titrated with 300 µM VP3.15, yielding only background-level signals. (B) Negative control ITC run in which buffer was titrated with buffer to establish the baseline noise level. (C) Positive control ITC run in which 30 µM ThiL was titrated with 400 µM thiamine monophosphate.

**
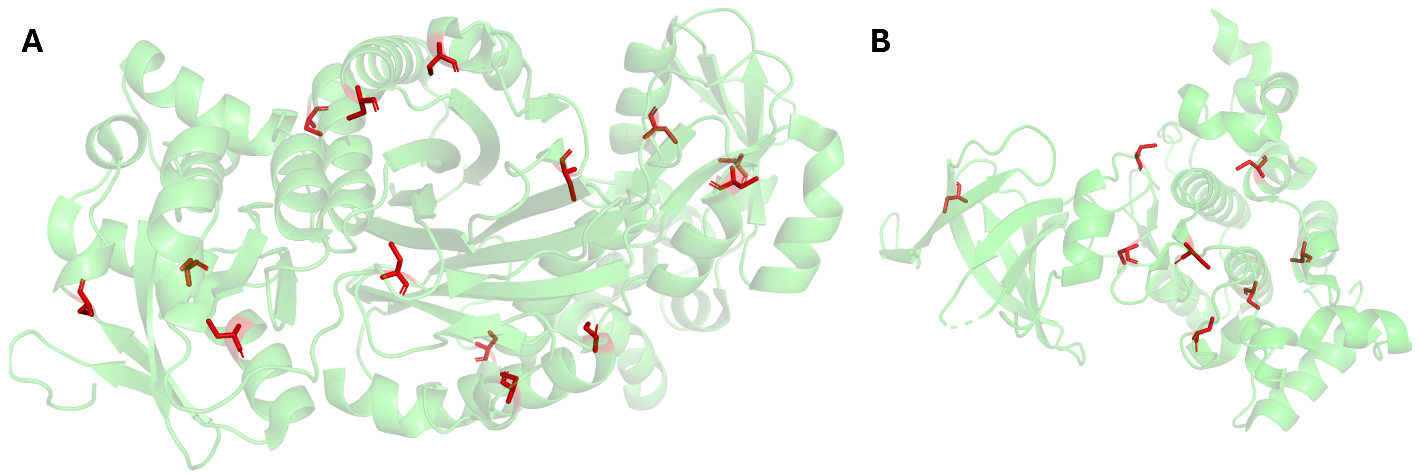
Supplementary Figure S4.** Free cysteine residues of (A) ThiL and (B) GSK3ß. Crystal structures of ThiL (PDB ID: 8YKS) and GSK3ß (PDB ID: 1I09) are displayed in cartoon with cysteine residues shown as red sticks. GSK3ß contains 9 cysteine residues while each ThiL protomer contains 7 cysteine residues. The first cysteine residue in GSK3ß resides in the N-terminal disordered loop and is therefore not displayed here.

**
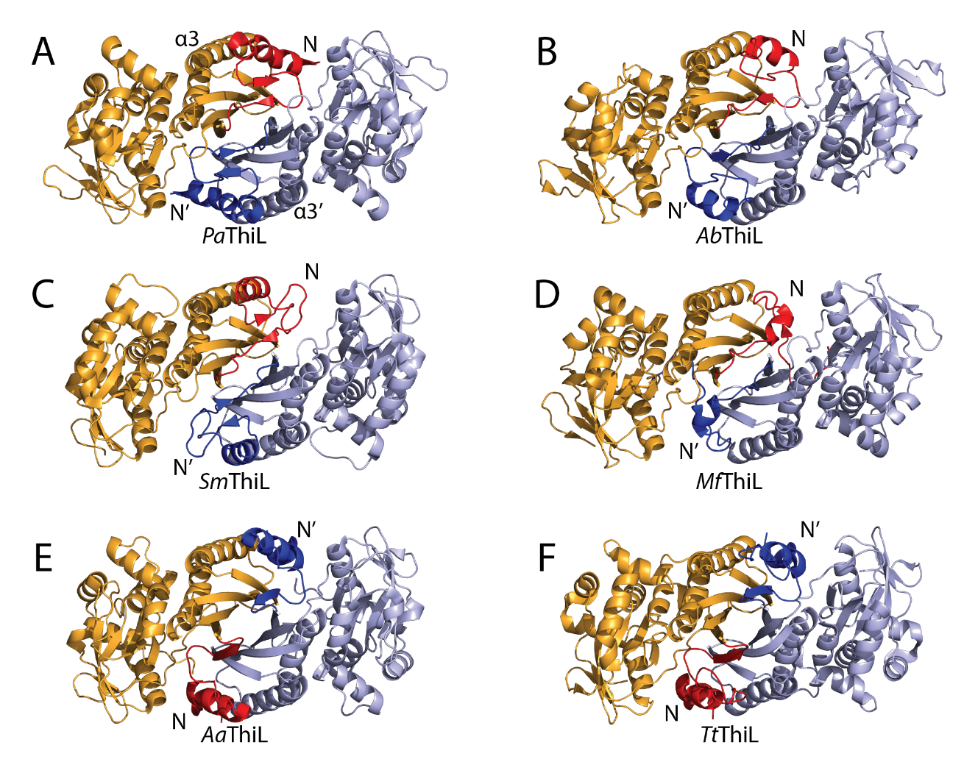
Supplementary Figure S5.** Different orientations of the N-terminal regions. Cartoon representations of *Pa*ThiL homologues viewed from the same angle as Fig. 2B. One ThiL protomer (left side from this viewing angle) is colored in orange and red (N-terminal region) while the other protomer on the right is colored in light blue and blue (N-terminal region). Note that residues 1-40 in *Pa*ThiL (A), residues 1-35 in *Ab*ThiL (B), residues 1-39 in *Sm*ThiL (C) and residues 1-36 in *Mf*ThiL (D) are all located next to helix α3 from the same (left) protomer. In contrast, residues 1-35 in *Aa*ThiL (E) and residues 1-39 in *Tt*ThiL (F) sit next to the helix α3’ from the other protomer via a domain exchange.

**
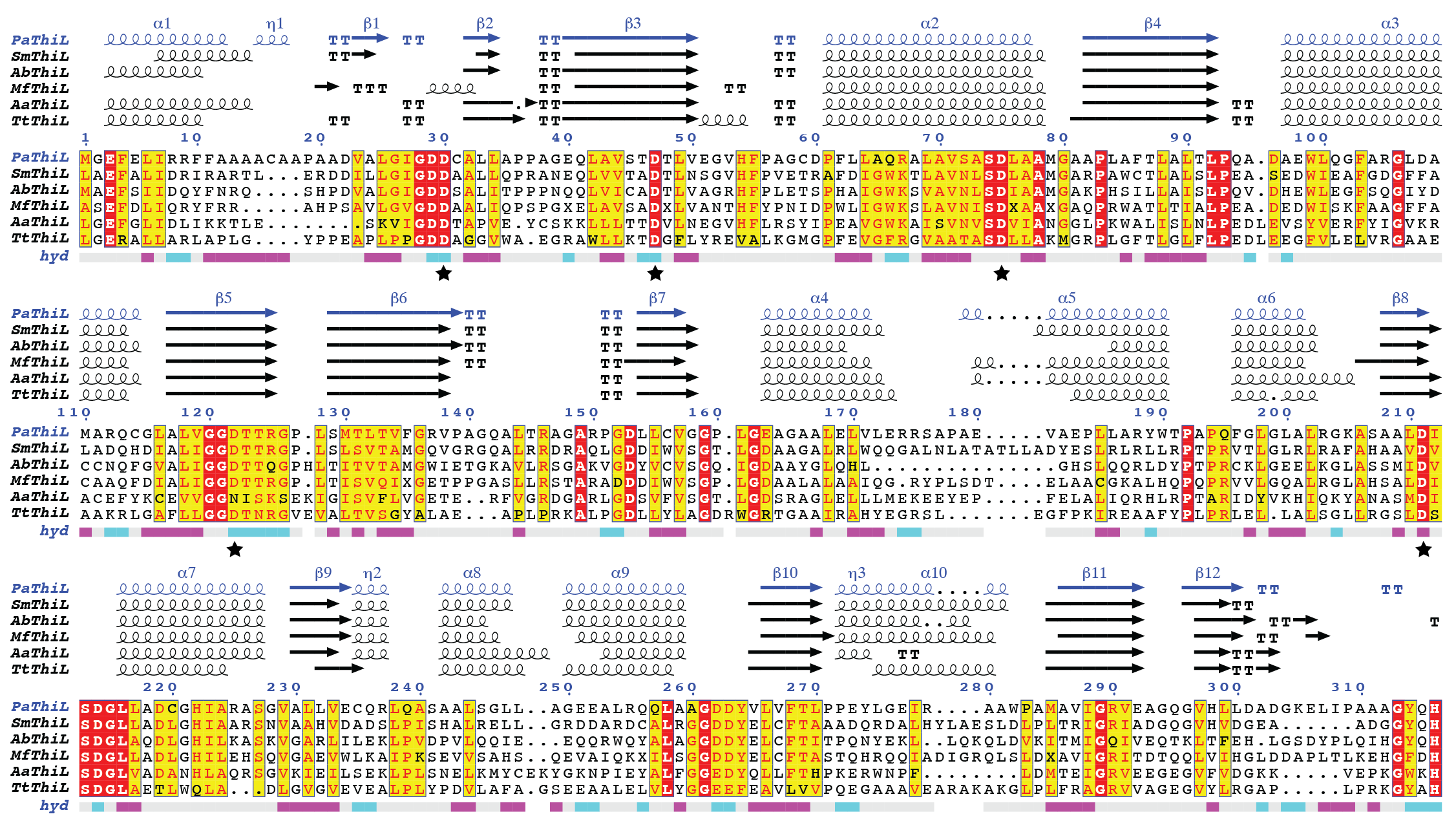
Supplementary Figure S6.** Structure-based sequence alignment of ThiL homologues. Secondary structure elements are largely conserved. Magnesium coordinating residues observed in *Pa*ThiL crystal structure is depicted by stars: Asp30, Asp47, Asp75 and Asp211 are strictly conserved, and Asp122 is conserved except in AaThiL. η: 310-helix, TT: β-turn and TTT: α-turn.

**
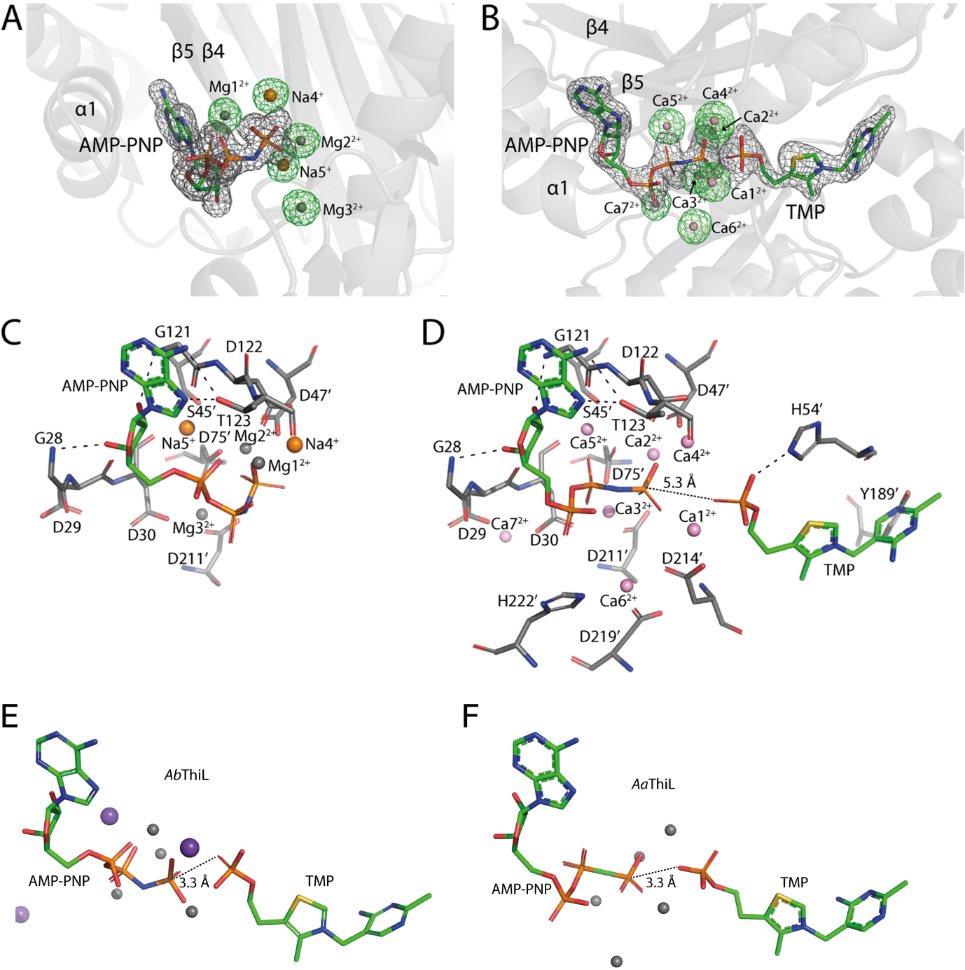
Supplementary Figure S7.** Close-up views of the PaThiL active site. (A) Binary complex. *Pa*ThiL is displayed as transparent cartoon. Omit difference Fourier maps are contoured at 4σ for both AMP-PNP (grey mesh) and metal ions (green mesh). (B) Ternary complex. Omit difference Fourier maps are also contoured at 4σ for AMP-PNP, TMP (grey mesh) and calcium ions (green mesh). (C) Coordination of AMP-PNP, Mg^2+^, and Na+ by the active site residues in the *Pa*ThiL binary complex. Hydrogen bonds are depicted by dashes. Water molecules are not shown for clarity. (D) Coordination of AMP-PNP, TMP, and Ca^2+^ by the active site residues in the *Pa*ThiL ternary complex. Distance from TMP α-phosphate to AMP-PNP γ-phosphate is 5.3 Å which is too far to initiate an in-line attack, resulting in a catalytically inactive. (E, F) Distances between the AMP-PNP γ-phosphate and TMP α-phosphate for *Ab*ThiL (E) and *Aa*ThiL (F) in the presence of magnesium. Purple sphere, K+; grey sphere: Mg^2+^.

**
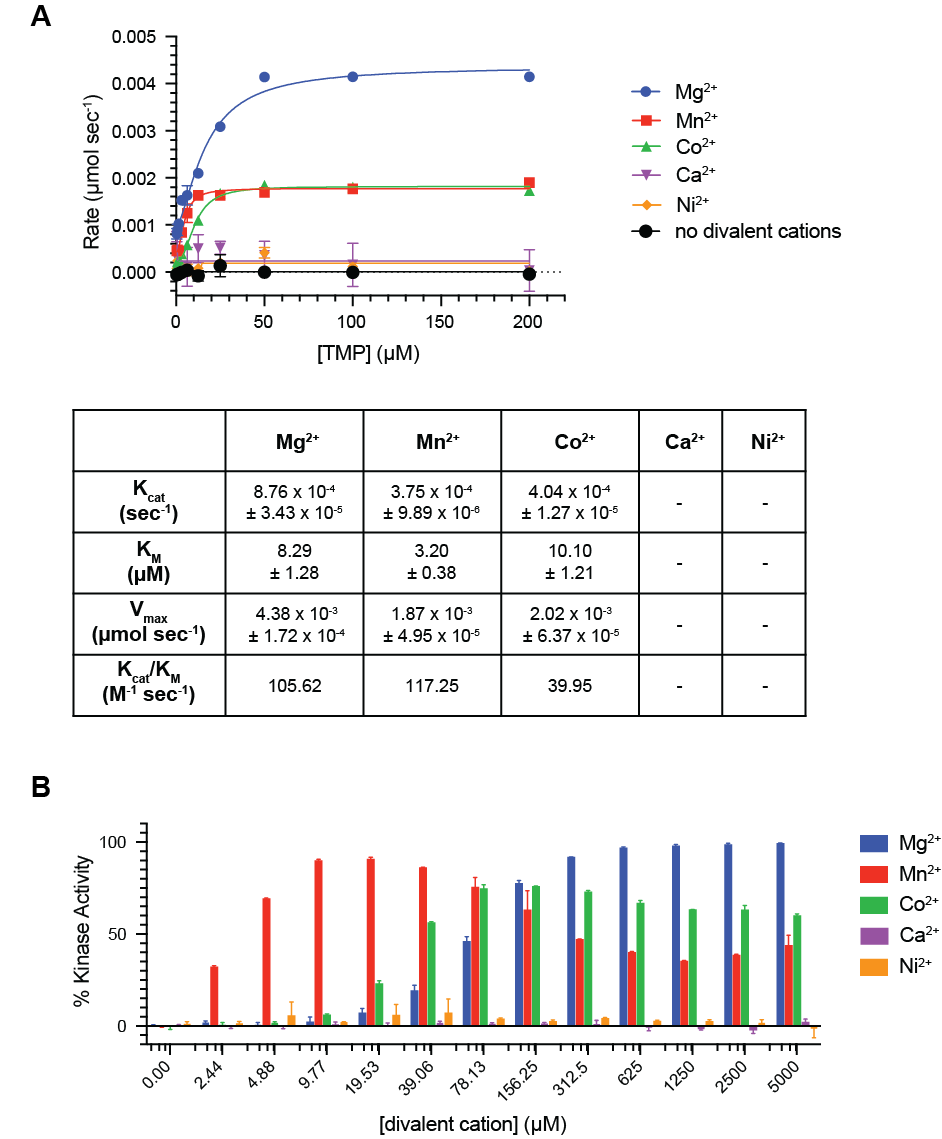
Supplementary Figure S8.** Metal dependency of PaThiL kinase activity. (A) Michaelis-Menten curves of *Pa*ThiL activity in the presence of 5 mM Mg^2+^, Mn^2+^, Co^2+^, Ca^2+^, Ni^2+^ or no divalent cations. Rates of reaction were determined by measuring ATP consumption (see Methods for details). Mg^2+^ provides the highest activity, while Ca^2+^ and Ni^2+^ provides none. (B) *Pa*ThiL activity in the presence of varying concentrations of divalent cations and a fixed concentration of substrates (50 µM ATP and 100 µM TMP). Mg^2+^, Mn^2+^, and Co^2+^ impart kinase activity to *Pa*ThiL in a concentration-dependent manner. Ca^2+^ and Ni^2+^ bestow no activity. All measurements were performed in triplicates and values are shown as mean ± standard deviation.

**Supplementary Table S1. Data collection and structure refinement statistics of *Pa*ThiL crystals.**

|  | **Binary complex of**  ***Pa*ThiL and AMP-PNP** | **Ternay complex of**  ***Pa*ThiL, AMP-PNP and TMP** |
| --- | --- | --- |
| **Data collection** | | |
| Wavelength (Å) | 0.9537 | 0.9537 |
| Resolution range (Å) | 44.21 - 1.80 (1.86 - 1.80)^a^ | 54.08 - 2.17 (2.25 - 2.17) |
| Space group | C222_1_ | P6_1_22 |
| Unit cell | | |
| *a, b, c* (Å) | 113.19 115.46 132.62 | 118.59 118.59 131.87 |
| *α, β, γ* (°) | 90 90 90 | 90 90 120 |
| Unique reflections | 80410 (7959) | 29549 (2895) |
| Multiplicity^b^ | 13.9 (14.0) | 33.2 (33.7) |
| Completeness (%) | 99.97 (100.00) | 99.98 (100.00) |
| I/sigma | 19.04 (2.46) | 17.37 (4.05) |
| R_merge_ (%)^c^ | 9.51 (102.20) | 23.79 (108.50) |
| CC_1/2_^d^ | 0.999 (0.823) | 0.998 (0.917) |
| CC*^e^ | 1 (0.95) | 1 (0.978) |
| **Refinement** | | |
| R_work_^f^ | 0.1840 (0.3411) | 0.1696 (0.2001) |
| R_free_^g^ | 0.1995 (0.3565) | 0.1761 (0.2143) |
| Average B-factor |  |  |
| Protein | 30.17 | 25.44 |
| Solvent | 37.71 | 34.16 |
| Ligands | 27.41 | 31.09 |
| RMSD |  |  |
| Bond lengths (Å) | 0.008 | 0.008 |
| Bond angles (°) | 1.08 | 0.97 |
| Ramachandran plot (%) |  |  |
| Favored | 98.43 | 98.34 |
| Allowed | 1.57 | 1.66 |
| Outliers | 0.00 | 0.00 |
| Rotamer outliers (%) | 0.00 | 0.48 |
| Clash score | 2.35 | 1.97 |
| PDB accession code | 8YKS | 8YKU |
| **Divalent cations** | Mg^2+^ | Ca^2+^ |
| ^a^Statistics for the highest-resolution shell are shown in parentheses. ^b^*N*_obs_/*N*_unique_. ^c^ R_merge_ = ∑\|*I*_j_ − < *I* > \|/∑*I*_j_, where *I*_j_ is the intensity of an individual reflection, and < *I* > is the average intensity of that reflection.  ^d^CC_1/2_—Pearson correlation coefficient between random half-datasets—*ρ_x_*,*_y_* = cov[(*x,y*)/(*σ_x_σ_y_*)] ^e^CC* = [2CC_1/2_/(1 + CC_1/2_)]^1/2^ ^f^*R*_work_ = Σ*_hkl_* \| \|*F*_obs_ \| −*k* \| *F*_calc_ \| \|/Σ*_hkl_* \| *F*_obs_\|. ^g^*R*_free_, calculated the same as for *R*_work_ but on the 5% data excluded from the refinement calculation. | | |

| Protein | PDB access code | Z score | r.m.s.d. (Å) | No. of aligned residues | Amino-acid sequence identity with *Pa*ThiL |
| --- | --- | --- | --- | --- | --- |
| *Ab*ThiL | 5CC8 | 38.4 | 1.7 | 288 | 39% |
| *Sm*ThiL | 6XEP | 36.9 | 1.9 | 293 | 45% |
| *Mf*ThiL | 3MCQ | 35.1 | 2.0 | 284 | 40% |
| *Aa*ThiL | 3C9U | 33.3 | 2.0 | 262 | 31% |
| *Tt*ThiL | 2YXZ | 29.4 | 2.1 | 260 | 32% |

**Supplementary Table S2.** Summary of DALI structural homology search results for *Pa*ThiL.
